# Human *in vivo* assessment of ketamine binding of the serotonin transporter—follow up at a higher dose

**DOI:** 10.3389/fnins.2025.1651016

**Published:** 2025-10-02

**Authors:** G. Schlosser, M. Murgaš, G. M. Godbersen, S. Reichel, L. Silberbauer, L. Nics, D. Winkler, T. Stimpfl, M. Hacker, S. Kasper, D. Rujescu, R. Lanzenberger, M. Spies

**Affiliations:** ^1^Department of Psychiatry and Psychotherapy, Medical University of Vienna, Vienna, Austria; ^2^Comprehensive Center for Clinical Neurosciences and Mental Health (C3NMH), Vienna, Austria; ^3^Department of Laboratory Medicine, Medical University of Vienna, Vienna, Austria; ^4^Department of Biomedical Imaging und Image-guided Therapy, Division of Nuclear Medicine, Medical University of Vienna, Vienna, Austria

**Keywords:** serotonin transporter (SERT), positron emission tomography (PET), ketamine, *in vivo*, neuroimaging

## Abstract

Ketamine is a rapid-acting antidepressant approved in the indication of treatment-resistant depression. As its clinical use expands, identifying underlying molecular mechanisms is essential. The serotonin transporter (SERT) is well known as a primary mechanism of several classes of monoaminergic antidepressants. Binding of ketamine to SERT has been observed *in vitro* and in animal studies with macaque monkeys using positron emission tomography (PET). We previously reported that a 0.5 mg/kg body weight dose of ketamine did not significantly bind to SERT in healthy human subjects assessed with PET but observed a positive trend between binding and ketamine plasma levels. Based on this finding, we hypothesized that a higher dose (0.8 mg/kg) would result in measurable SERT occupancy. Here, 10 healthy male participants were measured twice with [^11^C]DASB PET to test SERT occupancy following administration of 0.8 mg/kg body weight ketamine in four selected SERT rich regions amygdala, putamen, caudate and thalamus. Further, we implemented a bolus-plus-infusion radioligand infusion protocol and optimized the timing of the ketamine infusion. Contrary to our hypothesis, ketamine SERT occupancy did not significantly differ from zero, and the area under the curve of ketamine and norketamine plasma levels was not correlated with occupancy. These results suggest that even at doses up to 0.8 mg/kg, ketamine does not appreciably bind to SERT in humans, aligning with clinical observations that ketamine is routinely combined with serotonergic agents.

**Clinical trial registration:**

http://clinicaltrials.gov, identifier, NCT02582398. EUDAMED number, CIV-AT-13-01-009583.

## Introduction

Depression is the most common psychiatric disorder worldwide associated with substantial personal, societal, and economic burden (Smith, 2014). With the incidence of major depressive disorder rising steadily (Liu et al., 2020; Daly, 2022) the characterization and development of novel antidepressants becomes crucial.

Since the approval of esketamine nasal spray by the US Food and Drug Administration (FDA) and the European Medicines Agency (EMA) in 2019, ketamine has been implemented in clinical practice as a rapid-acting antidepressant for the treatment of treatment-resistant depression and suicidality. Early studies investigating ketamine’s antidepressant effects used intravenous administration of 0.5 mg/kg body weight racemic ketamine (Berman et al., 2000; Zarate et al., 2006). These studies showed robust antidepressant response rates of approximately 60% in patients suffering from treatment-resistant depression (Berman et al., 2000; Zarate et al., 2006; Aan Het Rot et al., 2010) and a significant reduction of suicidal ideation (Price et al., 2009, 2014). These findings have since been confirmed by meta-analytical evidence (Romeo et al., 2015; Bahji et al., 2021; Alnefeesi et al., 2022). To provide a more convenient route of administration and thus improve accessibility in outpatient settings, the esketamine nasal spray was developed, also demonstrating rapid and robust antidepressant effects in patients suffering from treatment resistant depression (Daly et al., 2019; Fedgchin et al., 2019; Ochs-Ross et al., 2020).

Ketamine has long been understood as a glutamatergic substance and noncompetitive open-channel blocker of the N-methyl-D-aspartate (NMDA) receptor. NMDA receptor antagonism remains a central mechanism of action for both antidepressant and dissociative effects (Anis et al., 1983; Davies et al., 1988; Zeilhofer et al., 1992; Zanos et al., 2018). Downstream effects of ketamine’s NMDA receptor antagonism include increased expression of hippocampal AMPAR subunits GluA1 and GluA2, as well as enhanced neuroplasticity (Zanos et al., 2023). However, NMDA receptor-independent mechanisms of ketamine and its metabolites (Zanos et al., 2016) and interactions with other neurotransmitter systems including dopamine, GABA, and glutamate have been described (Li et al., 2015; Kokkinou et al., 2018; Gerhard et al., 2020; Silberbauer et al., 2020).

Among these, ketamine’s serotonergic effects are of particular interest. In clinical practice, ketamine is routinely co-administered with serotonergic agents. Esketamine nasal spray was tested and approved for treatment-resistant depression in conjunction with a selective serotonin reuptake inhibitor (SSRI). Coadministration leads to no increase in serotonergic side effects (Daly et al., 2019; Fedgchin et al., 2019; Ochs-Ross et al., 2020). Additionally, there are case reports demonstrating that coadministration of ketamine and strong monoamine oxidase inhibitors like tranylcypromine appears safe (Bartova et al., 2015).

However, in animal studies ketamine’s effect on the serotonergic system appears to be key to its antidepressant properties. In a rat model of depression, serotonin depletion inhibits ketamine’s antidepressant effects (Gigliucci et al., 2013; du Jardin et al., 2016) while in monkeys intravenous application of high doses of ketamine leads to an increase in cortical serotonin (Yamamoto et al., 2013). Furthermore, elevated levels of cortical serotonin after bilateral injection of ketamine in mice correlate with the antidepressant effects (Pham et al., 2017). Thus, it had been hypothesized that ketamine’s rapid antidepressant effects may be linked to inhibition of the serotonin transporter (SERT; du Jardin et al., 2016), which is the primary point of action of many first-line antidepressants (Meyer et al., 2004). SSRIs like paroxetine show high occupancy of the serotonin transporter when measured with the highly selective SERT radioligand [^11^C]*N*, N-Dimethyl-2-(2-amino-4-cyanophenylthio)benzylamine ([^11^C]DASB; Meyer et al., 2004; The MICAD Research Team, 2004). Ketamine SERT binding was observed *in vitro* (Nishimura et al., 1998; Zhao and Sun, 2008) and in macaque monkeys with [^11^C]DASB positron emission tomography (PET; Yamamoto et al., 2013; Yamanaka et al., 2014). A previous [^11^C]DASB PET study in healthy subjects by our team revealed no significant ketamine SERT occupancy at the routine antidepressant dose of 0.5 mg/kg body weight but showed a trend toward an association between SERT occupancy and ketamine plasma levels (Spies et al., 2018). Though the study design was based on previous animal data (Yamamoto et al., 2013; Yamanaka et al., 2014), several confounding factors may have contributed to the lack of an observed effect. (1) Ketamine occupancy might only be present at higher doses. (2) Ketamine plasma levels fall sharply (Spies et al., 2018) making occupancy difficult to detect (3) ketamine metabolism is heterogeneous. The latter may be relevant if metabolites were to show affinity for SERT. Here, we assess ketamine and metabolite occupancy of the SERT in an optimized bolus-plus-infusion PET design.

## Materials and methods

### Subjects

Ten healthy male subjects aged 20 to 33 (mean ± SD 24.5 ± 3.7) were included. Subjects were screened for severe internal, neurological and psychiatric disorders. The screening visit included detailed medical history, physical examination, electrocardiography, blood pressure and a blood draw with a routine laboratory profile (hematology, clinical chemistry and coagulation). To screen for psychiatric disorders subjects were interviewed by an experienced clinician using the structured clinical interview for DSM-IV (SCID). Subjects with a history of drug abuse or intake of psychopharmacological medication were excluded. Furthermore, a urine drug test was performed at each visit. Subjects provided written informed consent and received financial reimbursement. The study was conducted according to the Declaration of Helsinki and approved by the Ethics Committee of the Medical University of Vienna (EK: 1643/2014).

### Inclusion criteria

1. Age 18–55
2. Somatic and psychiatric health
3. Capable of giving informed consent

### Exclusion criteria

1. Severe somatic illness
2. Psychiatric disorders as diagnosed by ICD-10 or DSM-IV, tested using the SCID
3. Clinically relevant alterations in blood draw, ECG, and somatic testing
4. Substance dependency disorder
5. Intake of psychopharmacological medication in the last 6 months
6. First degree relatives with Axis-1 disorder
7. MRI contraindications

### Study design

The study was designed as an open label study to optimize [^11^C]DASB PET methodology for assessment of ketamine SERT occupancy. In our previous study using 0.5 mg/kg body weight intravenous ketamine (Spies et al., 2018), PET quantification was limited by variability in timing between ketamine administration and the equilibrium of the radioligand infusion. Modifications in the present study aimed to improve the sensitivity for detecting potential ketamine SERT binding and to optimize the timing of the ketamine administration. Specifically, we implemented a [^11^C]DASB bolus plus constant infusion protocol to increase the amount of freely available radiotracer and thus improve the signal to noise ratio. A bolus plus constant infusion has been shown to be optimal to achieve rapid radioligand equilibrium in the thalamus and striatal regions (Gryglewski et al., 2017). Further, we minimized the temporal mismatch between ketamine plasma kinetics and radioligand distribution by beginning ketamine administration only after steady-state tracer binding was achieved, thereby reducing confounding from tracer disequilibrium.

Participants underwent two PET measurements using the highly selective SERT radioligand [^11^C]DASB. Baseline and ketamine conditions were measured within the same participants to reduce interindividual variability in ketamine SERT binding estimates. Furthermore, a fixed sequence of conditions was chosen to avoid carry over effects by ketamine since long acting neuroplastic effects of ketamine have been described (Aleksandrova and Phillips, 2021; Brown and Gould, 2024; Duque et al., 2025). During PET 1 no study medication was applied. During PET 2 0.80 mg/kg body weight of ketamine was administered intravenously. The PET measurements were carried out at an approximate time interval of 78.7 days (± 79.6 days), which was considered appropriate to ensure a sufficient washout of ketamine and its metabolites, and to reduce the likelihood of persisting neuroplastic effects (Aleksandrova and Phillips, 2021; Brown and Gould, 2024; Duque et al., 2025).

### Positron emission tomography

Ketamine SERT occupancy was measured with the highly selective SERT radioligand [^11^C]DASB using a GE Advance PET tomograph (GE Medical Systems, Wukesha, WI, at the Department of Radiology and Nuclear Medicine at the Medical University of Vienna). [^11^C]DASB was synthesized in house according to Solbach et al. (2004). For quality control radiochemical and chemical purity of [^11^C]DASB was assessed using high performance liquid chromatography. pH, isotonicity and residual solvents were evaluated using gas chromatography. Furthermore, in alignment with the regulations for radiopharmaceutical preparations from the European pharmacopeia sterility and potential endotoxines were tested. 15 MBq/kg body weight of [^11^C]DASB diluted in a maximum of 55 mL 0.9% saline solution were infused intravenously using a bolus plus constant infusion protocol over the course of 140 min, beginning 40 min prior to PET start (Gryglewski et al., 2017). Dynamic PET scans were acquired in three-dimensional (3D) mode. Following radioligand administration, 20 consecutive time frames of 5 min each were collected over a total scanning period of 100 min. Since approximately 40 min of the radioligand uptake occurred outside the scanner, scatter correction was applied to the emission data. The images were reconstructed into 35 contiguous slices using a 128 × 128 matrix, with each slice having a thickness of 4.25 mm. This yielded a final volume with a spatial resolution of 4.36 mm full width at half maximum (FWHM) at the center of the field of view.

### Magnetic resonance imaging

As part of the study two MRI measurements were performed. However, for this analysis only one structural MRI sequence was used for coregistration of PET data. The additional structural MRI scan was performed using a 3 Tesla PRISMA MR Scanner (Siemens Medical, 0.85×0.87 mm voxel size, 0.85 mm slice thickness, 230 slices).

### Region of interest selection

Region-wise analysis was performed due to the poor signal-to-noise ratio, with regions selected based on higher SERT abundance (thalamus, putamen, caudate, amygdala; Saulin et al., 2012) and quality characteristics for PET imaging including radioligand equilibrium after thorough examination of their respective time activity curves. In addition, cerebellar grey matter was utilized as reference region for quantification purposes (Kish et al., 2005; Walker et al., 2016).

### Study medication

0.80 mg/kg body weight of (R, S)-ketamine (Ketamine hydrochloride, Ketamin-hameln, 50 mg/mL ketamine ampoules, Hameln Pharmaceuticals GmbH) diluted with a maximum of 100 mL 0.9% saline solution was administered intravenously over the course of 50 min 60 min after PET start. This time frame was chosen to achieve the highest ketamine plasma levels during radioligand equilibrium. Due to a previous study revealing a potential dose dependant effect of higher subanaesthetic dosages (Spies et al., 2018) a dose of 0.8 mg/kg body weight was chosen to observe potential ketamine SERT binding.

### Ketamine metabolite assessment

Ketamine and norketamine plasma levels were determined during and after PET2. Blood samples were taken before ketamine application, 20, 40, 50, 55, 60, 70, 80, 110 and 130 min after tracer application. Plasma was centrifuged and stored at ≤ − 20 °C. Plasma levels were assessed using gas chromatography-tandem mass spectrometry (GC–MS/MS).

### PET data processing

PET scans were corrected for attenuation (using 5-min transmission scan with a ^68^Ge rod), decay and scatter and reconstructed to 20 frames (5 min long). Afterwards, PET data were corrected for head movement and were spatially coregistered to corresponding anatomical T1w MRI images, followed by normalization to MNI space. Time activity curves were extracted for each region separately. Visual inspection of TACs indicated a stable equilibrium between 95 and 135 min after tracer application. The average value of respective frames represents regional radioligand concentrations. These are further used to calculate the non-displaceable binding potential BP_ND_ based on the equilibrium method (Gryglewski et al., 2017).

### Statistics

Occupancy was calculated using the formula:


$$\text{Ocupancy}\;(\%)=(1-\frac{BP_{\mathrm{ND}}\;\mathrm{PET}2\;(\text{ketamine})}{BP_{\mathrm{ND}}\;\mathrm{PET}1\;(\text{control})})*100$$

A one-sample Wilcoxon signed-rank test was conducted to assess whether the median ketamine SERT occupancy significantly differed from zero. The area under the curve (AUC) for ketamine and norketamine plasma levels was calculated by generating a polynomial trendequation and subsequently taking the definitive integral of the trendequation. Missing values were assumed to lie on the polynomial trendequation between the previous and following Values. Correlation analyses between ketamine SERT occupancy and the AUC of ketamine and norketamine plasma levels were conducted using Spearman’s correlation coefficient. The significance level was be set to *α* = 0.05. Results were corrected for multiple testing via the Bonferroni-method.

## Results

Occupancy was calculated for the time span of 55 to 95 min after ketamine infusion for all subjects as visual inspection of ketamine plasma levels and radioligand TACs were suggestive of study drug and radioligand equilibrium.

**Figure 1:**
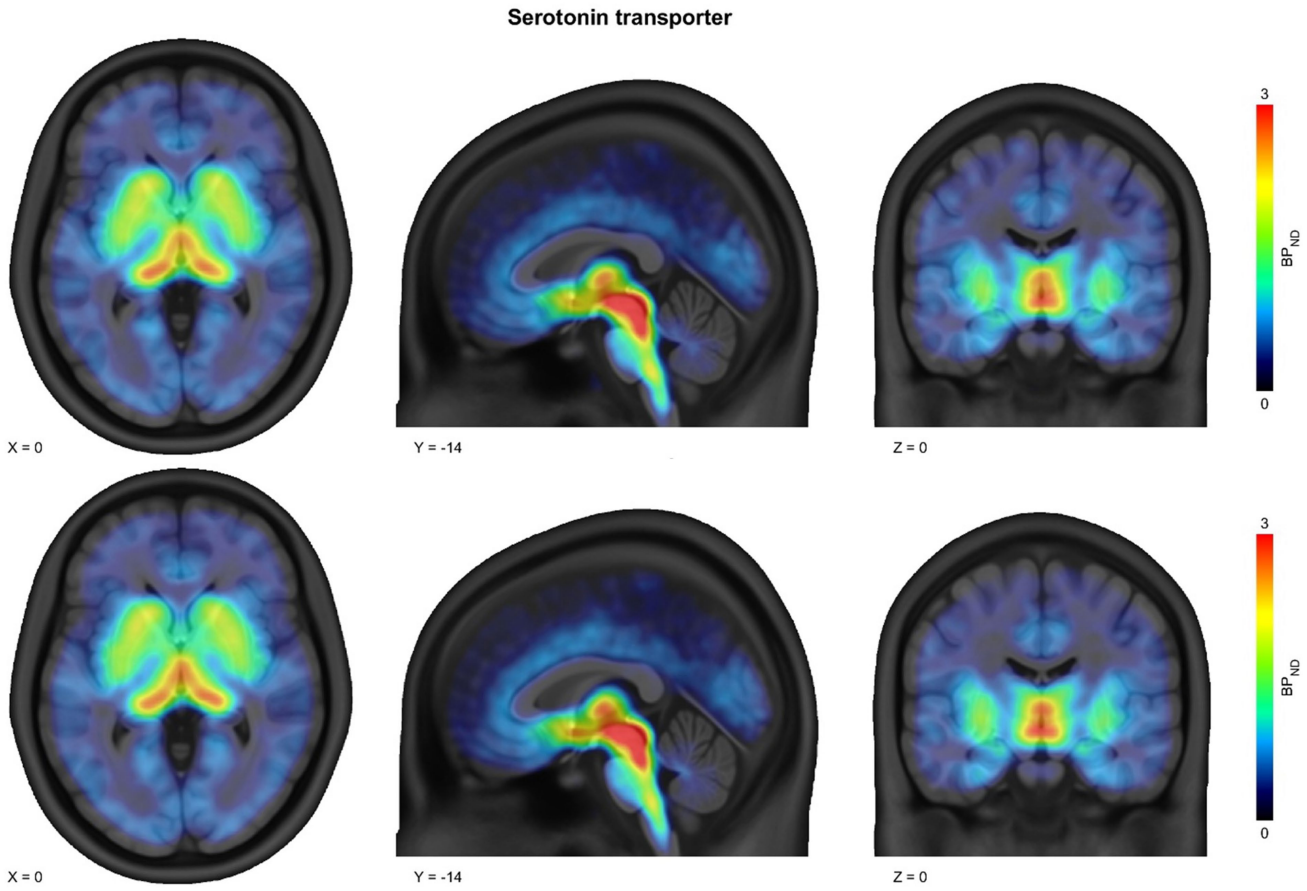
Parametric maps of [^11^C]DASB nondisplaceable binding potential (BP_ND_) at baseline (upper row) and after intravenous administration of 0.8 mg/kg ketamine (lower row). The images illustrate regional distribution of serotonin transporter (SERT) binding in the brain. Warmer colors on the color scale indicate higher BP_ND_ values, reflecting greater radioligand binding relative to nondisplaceable uptake. The maps show that visual differences between baseline and post-ketamine scans are minimal, consistent with the statistical analysis indicating no significant SERT occupancy by ketamine at this dose.

**Figure 2:**
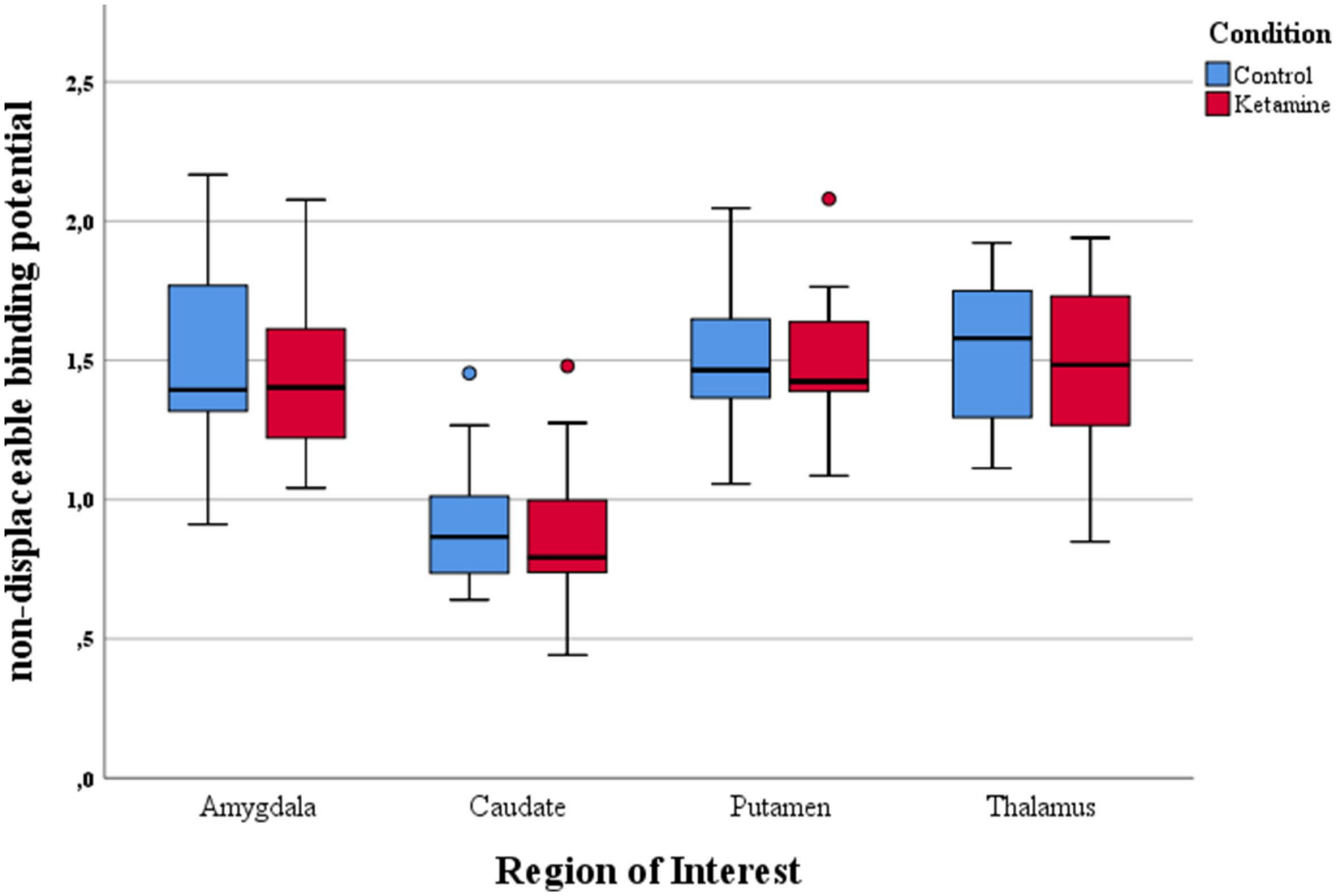
Boxplots of [^11^C]DASB nondisplaceable binding potential (BP_ND_) in the baseline condition (PET1) and following intravenous administration of 0.8 mg/kg ketamine (PET2). No systematic shift between conditions is evident, illustrating that ketamine administration did not alter SERT binding beyond normal variability.

**Figure 3:**
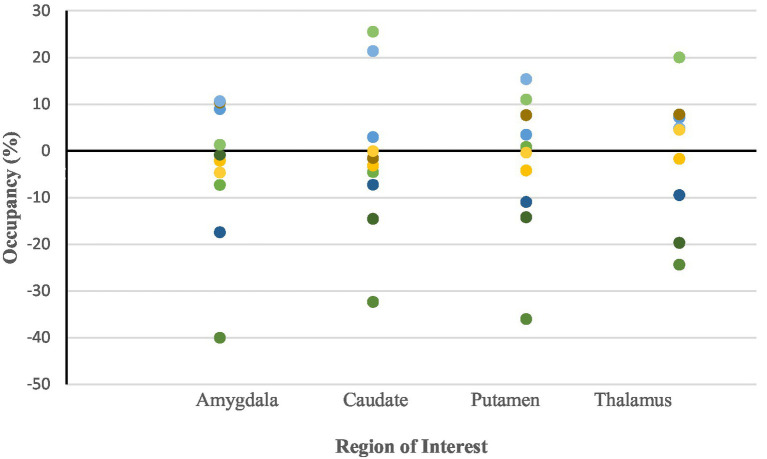
Dot plots of serotonin transporter (SERT) occupancy for each region of interest (ROI) following administration of 0.8 mg/kg ketamine. Occupancy is expressed as the percent reduction in [^11^C]DASB BP_ND_ from baseline to post-ketamine. Each dot represents an individual participant. A horizontal reference line at 0% is included to indicate the null hypothesis of no occupancy. ROIs include the amygdala, caudate, putamen, and thalamus, which are SERT-rich regions commonly investigated in antidepressant occupancy studies. Across all regions, occupancy values are close to zero and highly variable, suggesting that ketamine at this subanaesthetic dose does not produce measurable SERT binding *in vivo*.

Median occupancy over the group was at −1.51% (IQR = 19.11%), 0.23% (IQR = 20.27%), −2.39% (IQR = 16.64%) and, 4.6% (IQR = 19.36%) in the amygdala, caudate, putamen and thalamus, respectively (Table 1). Median values of the ketamine SERT occupancy of each ROI did not significantly differ from zero, as assessed with the Wilcoxon signed rank test (Figures 1–3). Specifically, the median occupancy did not differ significantly from zero in the amygdala (Z = −0.46, *p* = 0.65), putamen (Z = −0.76, *p* = 0.45), caudate (Z = −0.15, *p* = 0.88), or thalamus (Z = 0.15 *p* = 0.88).

**Table 1:** Descriptive statistics of ketamine serotonin transporter (SERT) occupancy for each region of interest (ROI).

Region of interest	Median (IQR)	Minimum	Maximum
Amygdala	−1.51 (19.11)	−40.05	10.62
Caudate	0.23 (20.27)	−32.38	15.32
Putamen	−2.39 (16.64)	−36.05	25.51
Thalamus	4.6 (19.36)	−24.38	19.99

AUCs were available for 9 out of 10 subjects.

Spearman’s rank correlation analyses were conducted to assess associations between ketamine SERT occupancy in four regions of interest (amygdala, caudate, putamen, nucleus accumbens) and the AUC of ketamine and norketamine plasma levels. For ketamine AUC, no significant correlations were observed: amygdala (*r*_s_ = −0.17, *p* = 0.67), caudate (*r*_s_ = 0.83, *p* = 0.83), putamen (*r*_s_ = −0.20, *p* = 0.61), and thalamus (*r*_s_ = −0.1, *p* = 0.80). Similarly, ketamine SERT occupancy in these regions was not significantly associated with norketamine AUC: amygdala (*r*_s_ = 0.28, *p* = 0.46), caudate (*r*_s_ = 0.05, *p* = 0.90), putamen (*r*_s_ = −0.18, *p* = 0.64), and thalamus (*r*_s_ = −0.67, *p* = 0.87).

## Discussion

In this analysis we examined cerebral ketamine SERT occupancy using an optimized bolus plus constant infusion PET protocol with the highly selective SERT radioligand [^11^C]DASB (The MICAD Research Team, 2004). We aimed to examine whether a dose of 0.8 mg/kg body weight of intravenous ketamine shows relevant SERT occupancy. Potential ketamine SERT binding at a dose of 0.8 mg/kg was hypothesizes based on a previous study with a dose of 0.5 mg/kg showing a trend association between SERT occupancy and ketamine plasma levels (Spies et al., 2018). Furthermore, [^11^C]DASB PET studies in macaque monkeys show relevant SERT occupancy at higher dosages of ketamine (Yamamoto et al., 2013; Yamanaka et al., 2014). Given that NMDA receptor–independent mechanisms of ketamine’s and its metabolites’ antidepressant effects have also been described (Zanos et al., 2016, 2018, 2023), we further aimed to test whether the AUC of ketamine and norketamine plasma levels correlate with SERT occupancy in humans.

Investigating binding of ketamine to the SERT is essential, as patients with treatment-resistant depression often suffer from severe somatic and psychiatric comorbidities and are treated with a variety of medications. This puts the patients at risk of drug interactions and undesired side effects since approximately 40% of approved medications bind to one or multiple serotonin receptors (McCorvy and Roth, 2015). Understanding ketamine’s molecular binding profile will aid in optimizing treatment safety and allow personalized interventions for a vulnerable population.

However, despite our implementation of bolus-plus-infusion protocol together with optimized dose and time-frame of ketamine infusion, occupancy values observed in preclinical animal studies could not be replicated, in alignment with our previous lower-dose study (Spies et al., 2018). Median occupancy values observed in our study were within the test–retest variability of [^11^C]DASB suggesting no relevant ketamine SERT occupancy (Kranz et al., 2015). Additionally, occupancy values observed differed greatly from those observed after clinical antidepressant dosages of SSRIs like paroxetine (Meyer et al., 2004). This suggests that the observed inhibition of the SERT by ketamine *in vitro* and in macaque monkeys may not be relevant in humans (Yamanaka et al., 2014).

A potential explanation is that the dosage of 0.8 mg/kg body weight used in this analysis is still insufficient to elicit relevant SERT binding. Both the 0.8 mg/kg and the 0.5 mg/kg used in our previous study have been found to be effective in treating depressive symptoms in clinical trials (Berman et al., 2000; Fava et al., 2020; Seshadri et al., 2024). However, a further dosage escalation may lack clinical relevance since the standard dose of 0.5 mg/kg body weight intravenous ketamine and doses above 0.5 mg/kg are similarly effective in treating depressive symptoms (Seshadri et al., 2024). Furthermore, doses of 1 mg/kg and more are associated with an increased risk of dissociation and blood pressure elevations (Fava et al., 2020).

Another cause for the lack of observed ketamine SERT binding may be the pharmacokinetics of ketamine and its metabolites. Ketamine undergoes extensive CYP metabolism, generating multiple active metabolites including norketamine, hydroxynorketamine, and dehydronorketamine, each with varying time courses and pharmacology (Zanos et al., 2018; Kamp et al., 2020). The timing of PET imaging in relation to peak plasma levels of these metabolites could therefore influence detectability of possible SERT interactions. In our data set, ketamine and norketamine AUCs were not related to SERT occupancy, although a more comprehensive analysis across additional time points and of further ketamine metabolites including hydroxynorketamine and dehydronorketamine would be needed to adequately capture pharmacokinetic–pharmacodynamic relationships.

Species differences in pharmacodynamics and metabolism may also explain discrepancies with animal studies (Yamamoto et al., 2013; Yamanaka et al., 2014). Elevated cortical serotonin levels after ketamine administration in animal studies (Yamamoto et al., 2013; du Jardin et al., 2016) may be caused by secondary mechanisms. These mechanism may include interactions with other neurotransmitter systems like GABA and glutamate (Zanos et al., 2016) and complex interactions with cortical serotonin receptors (Yamanaka et al., 2014).

We also considered regional specificity. We aimed at SERT-enriched regions (amygdala, caudate, putamen, and thalamus), but antidepressant action of SSRIs and possibly ketamine also involves cortical areas, e.g., the prefrontal cortex (Moda-Sava et al., 2019; Choi et al., 2021; Lee et al., 2023) and such regions as the bed nucleus of the stria terminalis (BNST; Cadeddu et al., 2016; Fiedler et al., 2021), which play a key role in mood regulation. While our present dataset had no frontal cortical regions included in the initial ROI selection, failure to find SERT occupancy in the selected striatal and limbic areas suggests that if there were any potential cortical effect, this too would be small compared with SSRI-induced levels of occupancy. Future studies can include cortex and more extended limbic regions in analysis to further deal with this problem.

These results however should be interpreted with care due to several limitations. Firstly, our sample size (*n* = 10) is limited. A small sample size is common in [^11^C]DASB PET studies investigating SERT binding of antidepressants (Meyer et al., 2004; Spies et al., 2018). However, the complex metabolism of ketamine must be considered. Ketamine is metabolized by a variety of enzymes with multiple active metabolites (Zanos et al., 2018). In accordance our dataset is not normally distributed with one outlier. We were also not able to correct for cytochrome-p-450 enzyme genotype or activity in our sample, which may contribute to variability (Desta et al., 2012; Rao et al., 2016; Zanos et al., 2016). A thorough investigation of the effects of ketamine metabolism on SERT occupancy would require a larger dataset, a broad range of dosages and potentially pharmacogenetic analyses. A further limitation is that our dataset only investigated healthy subjects therefore we cannot draw conclusions between ketamine SERT occupancy and treatment response to ketamine. Moreover, all our subjects were male. Sex specific effects of ketamine SERT occupancy may be relevant.

In conclusion, our study offers insights into ketamine’s effect on the serotonergic system in healthy subjects *in vivo*. We did not observe relevant binding of ketamine to the SERT after administration of a higher subanaesthetic dose of 0.8 mg/kg body weight ketamine despite optimization of ketamine dose and application times, as well as an optimized ROI selection. The occupancy values observed in our analysis differ greatly from those observed after application of minimal therapeutic dosages of SSRIs like paroxetine. Furthermore, a previous PET study by our team did not show relevant ketamine SERT binding as well. We hypothesize that inhibition of the SERT does not contribute substantially to ketamine’s clinical effects in humans. In accordance, ketamine is routinely combined with selective serotonin reuptake inhibitors and strong serotonergic agents like the monoamine oxidase inhibitor tranylcypromine with no increase in serotonergic side-effects (Bartova et al., 2015; Daly et al., 2019; Fedgchin et al., 2019; Ochs-Ross et al., 2020). Elevation of cortical serotonin levels after ketamine application described in animal studies (Gigliucci et al., 2013; du Jardin et al., 2016) may be a reflection of secondary mechanism.

## Data Availability

The raw data supporting the conclusions of this article will be made available by the authors, without undue reservation.
